# A comparative study of a nerve block therapy with and without a deeply inserted acupotomy applied to hyeopcheok points for lumbosacral radiculopathy: Safety, effectiveness, cost-effectiveness (a randomized controlled, two-arm, parallel study, pilot study, assessor-blind)

**DOI:** 10.1097/MD.0000000000028983

**Published:** 2022-03-04

**Authors:** Sooil Choi, Sukhee Park, Young-Soo Lim, Tae-Yong Park, Kwang-Sun Do, Sang Hyun Byun, Sang-Hoon Yoon, Jin-Hyun Lee

**Affiliations:** a Department of Anesthesiology and Pain Medicine, Catholic Kwandong University, International St. Mary's Hospital, Incheon, South Korea; b Institute for Integrative Medicine, Catholic Kwandong University International St. Mary's Hospital, Incheon, South Korea; c S-HEAL Pain and Korean Medicine Clinic, Seoul, South Korea; d Department of Applied Korean Medicine, Graduate School, Kyung Hee University, Seoul, South Korea.

**Keywords:** acupotomy, lumbosacral radiculopathy, nerve block therapy, pilot study, randomized

## Abstract

**Introduction:**

The prevalence of lumbosacral radiculopathy is estimated to be approximately 3% to 5% in patient populations. Lumbosacral radiculopathy is largely caused by a complex interaction between biomechanical and biochemical factors. Nerve block therapy (NBT) mainly treats lumbosacral radiculopathy by improving the biochemical factors, whereas acupotomy mainly focuses on improving the biomechanical factors. Therefore, it is thought that synergistic effects may be obtained for the treatment of lumbosacral radiculopathy when both NBT and acupotomy are combined. However, no study in China and Korea, where acupotomy is majorly provided, has reported the effects of such a combination treatment. Therefore, this study aimed to evaluate the safety, effectiveness, and cost-effectiveness of the concurrent use of a deeply inserted acupotomy and NBT for the treatment of lumbosacral radiculopathy.

**Methods/design:**

This is an open-label, parallel, assessor-blinded, randomized controlled trial, which will include 50 patients with lumbosacral radiculopathy. After patients voluntarily agree to participate in the study, they will be screened, and will undergo necessary examinations and tests according to the protocol. Those who satisfy the selection criteria will be randomly assigned to either the NBT + acupotomy or NBT groups in a 1:1 ratio. Both groups will undergo 2 NBTs once every 2 weeks from 1 week after the screening test. The treatment group will receive additional acupotomy twice a week for 4 weeks. The primary endpoint is the Oswestry Disability Index, whereas the secondary endpoints are the Numeral Rating Scale, European Quality of Life 5-dimension, McGill pain Questionnaire, Roland-Morris Disability Questionnaire, safety assessment, and economic feasibility evaluation. The measurements will be made at 0, 2, 4, and 8 weeks.

**Ethics and dissemination:**

This trial has received complete ethical approval from the Ethics Committee of Catholic Kwandong University International St. Mary's Hospital (IS20OISE0085). We intend to submit the results of the trial to a peer-reviewed journal and/or conferences.

## 1 Introduction

Although the literature lacks concise epidemiologic data, it is estimated by most reports that the prevalence of lumbosacral radiculopathy is approximately 3% to 5% in patient populations. Moreover, the condition constitutes a significant reason for patient referral to either neurologists, neurosurgeons, or orthopedic spine surgeons.^[1]^

Lumbosacral radiculopathy is the clinical term used to describe a predictable constellation of symptoms occurring secondary to mechanical and/or inflammatory cycles compromising at least one of the lumbosacral nerve roots.^[2]^ Patients can present with radiating pain, numbness/tingling, weakness, and gait abnormalities across a spectrum of severity. Depending on the nerve root(s) affected, patients can present with these symptoms in predictable patterns affecting the corresponding dermatome or myotome.

Lumbosacral radiculopathy is largely caused by complex interactions between biomechanical and biochemical factors.^[3]^ Biomechanical factors mainly cause continuous neuromuscular compression, which damages micro blood flow to the nerves and thereby activates C-fibers causing pain. Biochemical factors cause nerve damage by exposing phospholipase A2 to the surrounding nerves, leading to the production of various pathological substances that spread to the surrounding nerve roots, endings, and receptors to cause pain.

In nerve block therapy (NBT), a mixture of steroids and local anesthetics is administered to control pain through anti-inflammatory actions on inflammatory nerve roots and surrounding tissues.

Acupotomy is a special type of acupuncture that involves the addition of a scalpel function to the existing acupuncture needle. Since the tip of the acupotomy needle is a flat blade that can also function in synechotomy, the effects of both acupuncture and surgical treatment are expected from the procedure.^[4,5]^ Consequently, acupotomy treatment has been applied for various musculoskeletal diseases.^[6–11]^ Moreover, in China where acupotomy-related studies are actively being conducted, a total of 36 randomized controlled trials (RCTs) on lumbar disc herniation were published between 2006 and 2016,^[12]^ and 5 RCTs related to lumbar spinal stenosis were reported until 2017.^[13]^

However, in both China and Korea, there is no study on the effects of using both acupotomy and NBT as a combination treatment for lumbosacral radiculopathy.

Therefore, herein, we expect that the biochemical effects of NBT and the biomechanical effects of acupotomy will lead to a synergistic effect, and thereby propose to conduct a preliminary clinical study using the following protocol. The proposed study is expected to greatly contribute to the Korean and modern medicine combination treatment for lumbosacral radiculopathy and have important clinical implications.

### 1.1 Objective

The study aims to verify the hypothesis that the concurrent use of a deeply inserted acupotomy needle and NBT is more effective, safe, and cost-effective for lumbosacral radiculopathy than NBT alone.

### 1.2 Ethics and dissemination

This trial has received complete ethical approval from the Ethics Committee of Catholic Kwandong University International St. Mary's Hospital (IS20OISE0085). The findings in this study will be disseminated through peer-review journals and/or conference presentations.

## 2 Materials and methods

### 2.1 Trial registration

This study has been registered in the Clinical Research Information Service (CRIS, www.cris.nih.go.kr) (Trial registration number: KCT0006158; protocol version 1.3).

### 2.2 Study design

**Figure 1:**
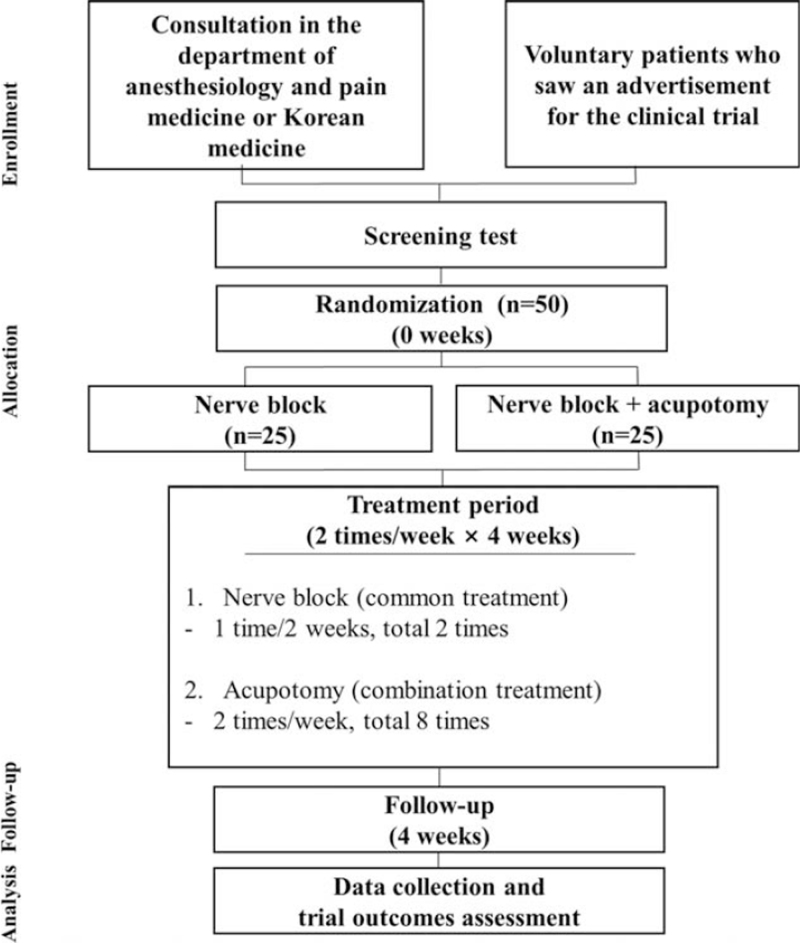
Study flow chart.

This study is designed as a randomized controlled, 2-arm, parallel, and assessor-blinded study. The study will be conducted at the Catholic Kwandong University, International St. Mary's Hospital, Incheon, South Korea. The clinical trial and the items to be examined in this study are presented in Figure 1 and Table 1. The patients will receive a full explanation of the details of the trial from the investigators. Through this procedure, if they agree to participate in the trial, a signed consent form will be provided.

**Table 1:** Clinical trial schedule and observation items.

Period		Screening^∗^ and Baseline	Treatment										Post Treatment	Early termination
Visit	Acupotomy + NBT group	Visit 1	Visit 2	Visit 3	Visit 4	Visit 5	Visit 6	Visit 7	Visit 8	Visit 9	Visit 10	Visit 11	Visit 12	Drop-out
	NBT group	Visit 1			Visit 2		Visit 3			Visit 4		Visit 5	Visit 6	
Day		0	1–3	4–6	7–8	9–11	12–14	15–17	18–20	21–22	23–25	26–28	56–58	
Written consent form		√												
Demographic survey		√												
Medical history, other medical history, and medication administration history		√												
Screening test^†^		√												
Selection/exclusion criteria		√												
Randomization (assignment of registration number)		√												
Physical examination and evaluation of vital signs		√	√	√	√	√	√	√	√	√	√	√	√	√
Medication administration history evaluation^‡^			√	√	√	√	√	√	√	√	√	√	√	√
Treatment	+ Acupotomy (combination treatment group)		√	√		√	√	√	√		√	√		
	NBT (all patients)				√					√				
Primary endpoint evaluation^§^		√					√					√	√	√
Secondary endpoint evaluation^||^		√					√					√	√	√
Exploratory efficacy evaluation^¶^													√	√^#^
Economic feasibility evaluation – cost evaluation^∗∗^		√					√					√	√	√
Adverse reaction monitoring			√	√	√	√	√	√	√	√	√	√	√	√
Economic feasibility evaluation – safety evaluation^††^			√	√	√	√	√	√	√	√	√	√	√	√

∗ One and 2 weeks are required from screening test to active treatment for combination treatment and NBT single treatment groups, respectively.

† Blood test [CBC 5 types, AST, ALT, ALP, albumin, BUN, protein, creatinine, total cholesterol, fasting glucose, RA, CRP, ASO, ESR, electrolyte 4 types (Na, K, Cl, T- CO_2_)]. It can replace test values performed within 8 weeks from screening.

‡ Concomitant medication history and adverse events monitoring are conducted at every visit for treatment and assessment.

§ ODI assessment.

|| Lower back and lower extremity pain (NRS) related to sacral neuropathy, quality of life (European Quality of Life 5 Dimension), Korean version of shortened MPQ, and Korean version of RMDQ.

¶ Additional NBT or surgical treatment within 4 weeks of the final procedure.

# Exploratory evaluation is not conduced for patients who have completed treatment early. For patients who dropped out of the study, exploratory evaluation is conducted after 4 weeks from dropping out (exploratory effectiveness evaluation for dropout participants can be conducted through telephone counseling instead of in-person visits).

∗∗ Evaluation of direct non-healthcare, direct non-healthcare, and lost productivity costs.

†† Those patients who reported adverse reactions will complete EQ-5D by recalling time of adverse events.

Patients who undergo 1 NBT and show continued symptoms (patient's subjective pain improvement is less than 50% after the procedure or the numeric rating scale (NRS) score being 5 or higher) will undergo screening at 1 week after the first procedure. For screening, demographic information, medical history, physical examination, vital signs, questionnaire survey, laboratory test, and selection/exclusion criteria will be evaluated. Randomization and group allocation will be performed at screening and baseline. Fifty patients will be enrolled and randomly divided into 2 groups. Patients who are judged suitable for the study after the screening test and voluntarily agree to participate will be randomly assigned to either the NBT single treatment group (usual care group) or NBT + deeply inserted acupotomy treatment group in a 1:1 ratio (Fig. 1). Thereafter, the randomly assigned patients will undergo primary and secondary outcome evaluations and economic evaluation.

The NBT + deeply inserted acupotomy group will receive 8 acupotomy treatments (at visits 2, 3, 5, 6, 7, 8, 10, and 11) for 4 weeks as well as additional 2 NBTs (at visits 4 and 9) for 4 weeks. Then, the group will be followed-up for 4 weeks after the intervention treatment. The standard care group will undergo two NBTs (at visits 2 and 4) for 4 weeks and will be followed-up for 4 weeks after the intervention treatment. Primary and secondary outcomes, economic evaluation, and assessment of drug administration history will be conducted at visits 1, 6, and 11, and at the end of follow-up. To evaluate safety, evaluation of adverse events, vital signs, and economic feasibility will be conducted at every visit from visit 2 to visit 12. Laboratory tests will be conducted only at screening.

Face-to-face evaluations will be conducted during the study period in parallel with the clinical trial. Then, face-to-face evaluations, distribution-type surveys, telephone surveys, or online meeting may be conducted after treatment period.

The coordinators of the trial will inform the patient of the next visit schedule for each visit and encourage participation. The clinical trial conductor will instruct the participant to ensure compliance with the treatment.

Only patients who have completed the informed consent form with full acceptance of the expected effects and predicted adverse reactions to the clinical trial interventions prior to commencement of the study may participate in the study. In the event of an accident or adverse event, appropriate measures will be taken according to the investigator's judgment and the participant will be compensated. Moreover, the study will be conducted ethically, in compliance with the principles of the Good Clinical Practice (GCP) guidelines and the revised version of the Declaration of Helsinki.

### 2.3 Sample size calculation

This is a practical clinical study to assess the effects NBT and deeply inserted acupotomy combination treatment on lumbosacral radiculopathy.

In a previous pilot RCT that compared the therapeutic effects of deeply inserted acupotomy and general acupuncture in lumbar disc herniation patients,^[14]^ considering a 20% drop-out rate, a total of 50 patients (25 each in the acupuncture and deeply inserted acupotomy groups) were included.

In another RCT that compared the effects of deeply inserted acupotomy and general acupuncture in lumbar disc herniation patients,^[15]^ a total of 40 patients (20 each in the acupuncture and deeply inserted acupotomy groups) were included.

The phase 1 preliminary clinical study that will be conducted aims to estimate the mutual effects of NBT and deeply inserted acupotomy combination treatment and assess whether the combination treatment is effective and safe compared with conventional treatment. Thus, the study must be conducted with a minimum number of patients within the limit that satisfies the study purpose. Currently, there is no previous study that has provided the same intervention. Therefore, based on previous studies that provided similar treatment interventions and involved disease groups as in our study, 20 patients were included in each group. Considering a dropout rate of 20%, a total of 50 patients (25 patients in each group) was set as the target sample size.

### 2.4 Intervention

#### 2.4.1 NBT

1. Drugs used in NBT (5–10 ml mixture of the following drugs)
  - Dexamethasone sodium phosphate 2.5 mg
  - 0.3% Mepivacaine
  - Hyaluronidase 1500 IU
2. Nerve block method
3. As long as there is no problem with safety based on the actual nerve block treatment method, the treatment site, dose, and method can be freely selected based on the discretion of the practitioner.
4. The type of NBT is the epidural block (epidural steroid injection) using the following approaches: interlaminar approach, transforaminal approach, caudal block, etc.
5. Number of treatments
6. Once every 2 weeks, a total of 2 times.

#### 2.4.2 Acupotomy therapy

1. Treatment tools
2. 0.7∗50 mm or 0.70∗80 mm-sized needles (Hansung, Korea) will be used in this study.
3. Number of treatments
4. Twice a week for 4 weeks, a total of 8 times after visit 2.
5. Treatment method
  (1) Application of an ice pack on low back skin for less than 20 minutes before the procedure to reduce pain.
  (2) Disinfection of the treatment area with alcohol, followed by povidone.
  (3) Performance of acupotomy on the disinfected treatment area. Consultation with experts to ensure safety during the procedure and conduction of ultrasound-guided acupotomy for standardized procedures. The detailed treatment areas and methods are shown in Supplementary Digital Content Appendix 1.
  (4) After the procedure, the treatment area is pressed for hemostasis using a sterile gauze for 3 minutes, and the area is covered with band-aids.
  (5) All patients must remain under observation for 15 minutes for any potential side effects and undergo education for prevention of infection in the treatment area and pain management before leaving.

### 2.5 Inclusion and exclusion criteria

#### 2.5.1 Inclusion criteria

The inclusion criteria are as follows:

1. adult men and women aged 18 to 85 years;
2. patients diagnosed with spondylosis with lumbosacral disc disorder (injury), spinal stenosis, or lumbosacral radiculopathy through radiological examination (magnetic resonance imaging or computed tomography) within 6 months prior to participation in the clinical trial (in case of patients suspected of lumbosacral radiculopathy but with no imaging records at the time of screening test, magnetic resonance imaging or computed tomography scans will be performed to check whether they meet the diagnostic criteria);
3. patients with symptoms related to lumbosacral radiculopathy, such as radiological pain, muscle weakness, and paresthesia of the lower extremities or those diagnosed with radiculopathy of the lumbosacral spine through a physical examination;
4. patients who will undergo NBT within 2 weeks after the start of the clinical trial, and patients whose subjective pain improvement will be less than 50% after the procedure or the NRS score will be 5 or higher;
5. patients who can read, understand, and answer the symptom questionnaire; and
6. patients who volunteer to participate and give their written consent while agreeing to the clinical trial plan and follow-up.

#### 2.5.2 Exclusion criteria

The following patients would be excluded from participating in the trial:

1. patients with a history of spinal surgeries, such as lumbar spine intrametallic fixation and spinal fusion, or those who have undergone past spinal surgery but continue to have related pain;
2. patients with cauda equina syndrome or motor paralysis and neurological symptoms, which are expected to be difficult to treat with conservative therapy and require surgical treatment;
3. patients taking drug treatments, such as strong opioid therapy, for pain control;
4. Patients who received acupotomy treatment within 2 weeks before the start of the clinical study (inclusion will be permitted despite treatment at another institution within the relevant period for reasons different from the indications defined for this clinical trial at the discretion of the clinical trial practitioner);
5. patients who have experienced side effects or hypersensitivity reactions after NBT in the past;
6. patients with acupuncture hypersensitivity, metal allergy, severe atopy, keloid skin, and other skin sensitivities;
7. patients with hemophilia;
8. patients who are taking drugs that can cause hemostasis, such as anticoagulants, antiplatelet drugs, aspirin, etc., wherein it is not possible to stop the drug during the clinical trial period, according to the judgment of the clinical trial practitioner;
9. patients who participated in other clinical studies within 30 days prior to the screening of the current clinical study and received investigational drugs, including placebos;
10. patients with psychotic disorders, alcoholics, and drug addicts;
11. pregnant women, lactating women, and women of childbearing potential who are not willing to use contraception during the clinical trial; and
12. patients who are judged to be inappropriate for participation in clinical trials by the clinical trial practitioner.

### 2.6 Recruitment, randomization, blinding, and nonblinding

Advertisements for patient recruitment will be posted on the Catholic Kwandong University, International St. Mary's Hospital's bulletin boards, along with posters, banner advertisements, and internet cafes. All advertisements will be run after institutional review board (IRB) approval.

Individuals who provide written consent to participate in the study will be assigned a screening number, according to the order of the outpatient visit at screening. Unblinded sub-investigators, who do not influence the research results and analysis, will refer to the randomization list that has been prepared by a commissioned professional statistician, and assign participation numbers. Participants will be allocated to the NBT group and the acupotomy + NBT group in a ratio of 1: 1. A block randomization table will be generated by an independent statistician and patients will be assigned to one of 2 groups according to the randomization table. Screening numbers, randomization numbers, and initials assigned to each participant will be used as subject identification codes to identify the participant until the end of the clinical trial.

The NBT used in this study is not a clinical investigational product and does not require allocation concealment, since it is administered to all groups. Moreover, it is impossible to blind the investigator and the participant in each group due to the nature of the study intervention, which is based on the contact between the patient and the medical staff. Instead, the investigator maintains blinding to prevent bias in evaluating the participant. After the clinical trial is completed, all clinical report forms (CRFs) will be collected, the database process terminated, and unblinding performed, if required for statistical analysis. Moreover, if it is deemed necessary to confirm the subject group due to an emergency situation during the clinical trial, the randomization code will be disclosed only to the participant, according to the principal investigator's discretion. If it is not possible to consult with the principal investigator and the situation is urgent, the randomization code of the subject will be disclosed after consultation with the randomization code officer. If unblinding is performed, the documentation of the reason for the unblinding will be retained, and the participant with the disclosed randomization code will not be able to continue the trial.

### 2.7 Rescue therapy and concomitant medications

If severe unbearable pain is observed at the time of visit for screening, limited anti-inflammatory analgesics and muscle relaxants, excluding opioid analgesics and anesthetics, are permitted at the discretion of the clinical investigator providing the NBT (Medications for the digestive system are also allowed to reduce the side effects of rescue medications.). If rescue medications are used, the date and dosage of administration must be recorded in the patient journal.

The types of available rescue medications include skeletal muscle relaxants, anti-convulsant, anti-inflammatory drugs, analgesics, neuralgia drugs, anti-depressants, nonopioid analgesics, antipyretics, weak opioids, and digestive medications.

### 2.8 Study outcomes

#### 2.8.1 Primary outcome

Functional assessment will be conducted using the Korean Version of Oswestry Disability Index (ODI)^[16]^ at 0 (screening test), 2, 4, and 8 weeks compared with that before study participation will be investigated. However, considering the cultural characteristics of Korea, item number eight on sex life will not be asked.

#### 2.8.2 Secondary outcome

1. NRS
  The patient selects a number that corresponds to the pain level^[17]^ (NRS-11 that evaluates pain from a score of 0–10 will be used). In this study, changes in pain level will be assessed at 0 (screening test), 2, 4, and 8 weeks and compared with that before study participation. Low back pain and lower extremity pain will be separately evaluated.
2. European Quality of Life 5 Dimension (EQ-5D)
  Changes in quality of life will be assessed at 0 (screening test), 2, 4, and 8 weeks and compared with that before study participation. EQ-5D evaluates 5 domains: mobility, self-care, usual activity, pain/disability, and anxiety/depression. It is widely used to assess quality of life.^[18]^
3. McGill Pain Questionnaire (MPQ)
  The Korean version of MPQ^[19]^ will be used to assess changes in pain at 0 (screening test), 2, 4, and 8 weeks and compared with that before study participation.
4. Korean version of Roland–Morris Disability Questionnaire (RMDQ)
  The Korean version of RMDQ^[20]^ will be used to assess changes in pain at 0 (screening test), 2, 4, and 8 weeks compared with that before study participation.

#### 2.8.3 Exploratory effectiveness evaluation

1. Additional NBT or surgery for lumbosacral radiculopathy: All patients will be assessed if they underwent any additional NBT or surgical treatment related to lumbosacral radiculopathy on the last day of visit and at 4 weeks after the end of treatment. This assessment will be not conducted on those whose symptoms have improved and ended the study earlier. For patients who dropped out in the middle of the study, the assessment will be performed at 4 weeks after dropout. However, exploratory effectiveness evaluation for dropout participants can be conducted through telephone counseling instead of in-person visits.
2. Early termination of treatment: Patients whose symptoms have improved during the study, who request to terminate treatment early, and who no longer require treatment at the discretion of the investigators will be separately evaluated for each group.
3. Rescue medication usage: The number of uses, dosage, type, and administration point of rescue medications used between the clinical trial period and final evaluation period will be compared between the 2 groups.

#### 2.8.4 Evaluation of responders and nonresponders

1. Patients will be divided into groups of responders and nonresponders based on the NRS with a minimal clinically important difference (CID) of 2.5 for chronic low back pain.^[21]^ If the NRS score decreases by 2.5 or more compared with that at screening, the patient will be considered as a responder, and vice-versa.
2. Patients who terminated their treatment earlier after improvement of symptoms will be included in the responder group.

#### 2.8.5 Safety and adverse events monitoring

1. Physical examination will be conducted and vital signs will be collected at every visit after randomization. The results of the physical examination and vital signs will be reviewed to determine the normality and abnormality of each examination.
2. Patient Reported Outcome-Common Terminology Criteria for Adverse Events (PRO-CTCAE)
  CTCAE evaluated by the medical staff involves assessment of symptoms by the clinician, and this may lead to the omitting or underestimation of side effects experienced by the patients. Therefore, the importance of patients directly explaining their overall health status is increasingly emphasized. In this study, the PRO-CTCAE^[22]^ questionnaire will be used to assess patient safety. It is expected that the main side effect of neuropathic pain treatment will be aggravation of pain. Thus, terms of pain-related symptoms in the PRO-CTCAE will be selected and used to construct a questionnaire. The assessment will be conducted at 2, 4, and 8 weeks.3. Evaluation of objective and subjective symptoms as well as adverse reactions will be conducted at every visit after randomization.
  - The investigator must record all adverse reactions and concomitant medications used during the clinical trial.
  - Adverse reactions and concomitant medications will be recorded in the case record form. For adverse reactions, the symptoms, signs, duration (start date/end date), severity, outcome, seriousness, causal relationship with the intervention, and actions taken for the reaction will be recorded.
  - Symptoms or signs that are present before participation in the study will not be recorded as adverse reactions. However, changes in the frequency, severity, and scope of the symptoms after participation in the study will be recorded as adverse reactions.
  - Additionally, the name of ingredients, dosage, administration period, and reasons for administration of concomitant medications will be recorded in detail.

#### 2.8.6 Evaluation of economic feasibility

1. Demographic characteristics
  - Marital status, occupation, education level, religion, household income, etc., will be recorded by the patients at the first visit.
2. Effectiveness
  - EQ-5D will be recorded by the patients at 0, 2, 4, and 8 weeks.
3. Safety:
  - EQ-5D: Patients who reported adverse reactions at all visits after randomization will self-report the time of adverse events through memory recall.
4. Cost
  - Direct medical cost will be asked by the investigator at 0, 2, 4, and 8 weeks, and the patients will report in the CRF.
    - Cost of deeply inserted acupotomy: data on institutional cost
    - Expenses related to inpatient, outpatient, and emergency room treatment when visiting a medical institution for lumbosacral radiculopathy
    - Expenses for rescue/concomitant medications
    - Expenses for other instruments, treatment materials, and home remedies
  - Direct nonmedical cost will be asked by the investigator at 0, 2, 4, and 8 weeks, and the patients will report in the CRF.
    - Transportation fee
    - Nurse fee
    - Patient time expenditure cost
  - Productivity loss cost: the patients will self-complete the iMTA Productivity Cost Questionnaire at 0, 2, 4, and 8 weeks
    - iMTA Productivity Cost Questionnaire

#### 2.8.7 Early termination or dropout

##### 2.8.7.1 Dropout criteria

1. If the patient is under prescription of the following medications that are expected to affect the safety and effectiveness of NBT and acupotomy, the medications cannot be taken from the time of screening until the completion of the study.
  ① Concurrent use of medications that are prohibited for use with medications used for NBT.
    Medications that are likely to cause serious side effects in drug interaction studies.
    Medications whose mutual use is restricted with disodium dexamethasone injection: asunaprevir, daclatasvir, dronedarone hydrochloride, eliglustat, grazoprevir, ibrutinib, nilotinib hydrochloride monohydrate, olaparib, osimertinib, palbociclib, rilpivirine, rivaroxaban, ticagrelor, ceritinib, ribociclib, venetoclax, elbasvir, BCG strain, cobimetinib, cicletanine, etc. (Other medications used for NBT do not cause side effects of serious grade in drug interaction studies)
  ② Strong opioid, local anesthetic administered by another physician than the one for this clinical study
    However, anti-inflammatory analgesics and muscle relaxants prescribed for necessary causes at the discretion of the clinician can be administered^[13]^ (See rescue medications).
  ③ Medications that may pose a risk to the patient or cause bias in the results as judged by the clinical investigator
2. The patient requests to discontinue interventions or withdraws consent for participation
3. The patient is judged to be unfit to continue in the study due to serious adverse reactions/medication reactions
4. Serious protocol violations such as inclusion/exclusion criteria are newly discovered during the study
5. Patients in the NBT and acupotomy combination treatment group who do not meet the criteria for early termination but have not conducted 6 or more of the 8 planned acupotomy treatments
6. Patients who do not meet the criteria for early termination but fail to conduct even one of the planned NBT
7. Patients who are judged to be unfit to continue in the study for other reasons at the discretion of the clinical investigator

##### 2.8.7.2 Criteria for early termination

1. Patients whose symptoms have improved during the study, who request to terminate early, and who no longer require treatment at the discretion of the investigators can terminate the study earlier than planned.
2. Patients who have terminated the study early are not classified as withdrawn/discontinued patients and are considered to have complied with the study treatment.

#### 2.8.8 Data collection, access, and management

All information regarding the patient is anonymized through initial processing, and all investigators are obliged to maintain confidentiality of the results. The source document is registered immediately when the data are collected, and it will be recorded in the CRF. All the documents of the trial will be kept safe, and only those who have been approved by the principal investigator will have access to all data related to the trial.

Data management of this clinical trial will be conducted in accordance with the standard working guidelines of the Catholic Kwandong University Clinical Research Center, and other matters not specified in the protocol will be conducted following the International Council for Harmonization of Technical Requirements for Pharmaceuticals for Human Use guideline for GCP and Korea-GCP standards.

### 2.9 Statistical analysis

For efficacy analysis, the intent-to-treat (ITT) group will be mainly analyzed, and secondary analysis will be done on the per-protocol (PP) group. Safety analysis will be performed on the ITT group patients who undergo at least one procedure after randomization. The ITT group will include all patients who have been randomly assigned, undergo at least one follow-up, and are eligible for efficacy assessment. PP group will include patients who satisfy the following conditions:

1. patients who satisfy both selection and exclusion criteria;
2. those who have completed the pre-determined minimum number of acupotomy (6 out of 8 sessions); and
3. those whose symptoms were improved and who satisfied treatment completion criteria

1.
**Demographic information and pre-treatment characteristic analysis method**
  Descriptive statistics will be conducted for demographic information and characteristics. Continuous data will be expressed using the number of observations, mean, standard deviation, median, 25% quantile, and 75% quantile. Categorical data will be expressed using frequency and percentage. For continuous data, independent *t*-test will be conducted to test the mean difference between groups if normality assumption is satisfied. If normality assumption is not satisfied, Wilcoxon's rank sum test will be conducted. Chi-Squared test will be conducted to test the mean difference between groups. If more than 25% cells have an expected frequency of less than 5, Fisher's exact test will be conducted.
2.
**Efficacy evaluation**
  In the efficacy evaluation, ITT will be treated as the main analysis group, and PP sets will be treated as the secondary analysis group. All statistical analysis will be conducted as two-sided tests with a significance level of 5%. Two-sided 95% confidence interval will be presented. Descriptive statistics will be presented for each treatment group and visit. Descriptive statistics and 95% confidence intervals will be presented for differences between the baseline and each visit.

Among efficacy evaluation variables, missing values of continuous variables will be analyzed using baseline observation carried forward correction.

A. Analysis of primary endpoints

For symptoms of lumbosacral radiculopathy at each measurement time point, descriptive statistics (number of patients, mean, and standard deviation) of the ODI score and changes in ODI at the end of treatment (4 weeks) and follow-up (8 weeks) compared with the baseline will be presented.

Paired *t*-test or Wilcoxon's signed ranked test will be conducted for intra-group changes in ODI at the end of treatment (week 4) compared with the baseline (0) depending on the satisfaction of normality. An analysis of variance model that includes changes in ODI at the end of treatment compared with the baseline as effects and baseline ODI as covariates will be used to evaluate the effects in the treatment group compared with the control group. Differences in least-square mean between treatment and control groups will be calculated, and two-sided 95% confidence interval and *P* value for the differences will be presented.

B. Analysis of secondary endpoint

- Changes in ODI at follow-up (8 weeks) compared with baseline
- Assessment of subjective pain intensity in patients with lumbosacral radiculopathy
  - Lumbosacral radiculopathy pain will be divided into lower back and lower extremity pain using NRS, MPQ, and K-RMDQ
- Assessment of quality of life related to lumbosacral radiculopathy
  - Evaluation of overall quality of life using EQ-5D. The same analysis methods for primary endpoints will be used to analyze secondary endpoints

C. Exploratory efficacy evaluation

- Evaluation related to additional NBT or surgery after completion of the study

The rate of additional treatment will be evaluated by assessing additional NBT or surgery performed for pain control within 4 weeks after the end of treatment. Chi-Squared test or Fisher's exact test will be performed to compare the rate of additional treatment and surgery between the groups.

**Rate of additional procedures = (patients who underwent additional procedures or surgeries/patients who completed the entire study)∗100**

- Assessment of treatment early termination rate

Treatment early termination rate will be calculated for each group for patients who satisfied the criteria for early termination of treatment. Chi-Squared test or Fisher exact test will be performed to compare the rate of early termination of treatment between the groups.

**Rate of early termination = (patients who completed the study early/patients who completed the entire study)∗100**

- Analysis of rescue medication use

Independent t-test or Wilcoxon's signed rank test will be conducted to compare the number and dose of use according to different types of rescue medication between the groups.

D. Classification and post hoc analysis of responders and nonresponders

- Definition of responders

(1) Those with a minimal CID decrease of NRS 2.5 from the baseline to the date of evaluation. Therefore, responders will be defined as follows. For example, pain reduction of greater than NRS 2.5 will be defined as CID, and CID will be used to divide patients into responders and nonresponders.
(2) Using the previously described criteria, patients who had improved symptoms and completed the study early will be considered as responders.

- Post hoc analysis

Adequate post-hoc analysis will be conducted by a statistician for differences in treatment results for each treatment group using the responder and non-responder criteria.

**3. Safety assessment**

Descriptive statistics will be conducted to analyze PRO-CTCAE questionnaire items, number of adverse events, number of patients with adverse events, severity of events, and causal relationship of adverse events with the intervention for each dose group. Nonparametric methods may be used if necessary.

Vital signs, physical examination results, and subjective complains of patients will be comprehensively reviewed to perform statistical analysis on items that are considered clinically significant by the researcher. Continuous variables will be presented using the number of observations, mean, standard deviation, median, and minimum/maximum values, and differences in the mean between the 2 groups will be analyzed using paired *t*-test or Wilcoxon's signed rank test. Chi-Squared test or Fisher Exact test will be conducted for analysis of adverse events. Moreover, clinically significant changes will be compared between groups through generalized estimating equation analysis to assess statistical significance.

**4. Economic feasibility evaluation**

Economic feasibility will be evaluated on patients who participated in the clinical trial at least once since visit 2. The economic endpoint to assess cost-effectiveness will be incremental cost-utility ratio, which will be calculated based on the difference in cost and quality-adjusted life year between the experimental and control groups. Quality-adjusted life year will be calculated using EQ-5D measured at the time of visit.


$$\text{ICUR}=\frac{\text{Total Cost}\;(\text{treatment group})-\text{Total Cost}\;(\text{control group})}{\text{Quality-adjusted life year}\;(\text{treatment group})-\text{Quality-adjusted life year}\;(\text{control group})}$$

A. Time horizon

Basic time horizon for analysis will be 4 or 8 weeks. If long-term follow-up beyond 8 weeks is required, the Markov model will be used. Transition probabilities will be estimated based on clinical data collected over 8 weeks. In addition, if the time horizon exceeds 12 months, the unit of cost will be in Korean currency unit (won) for specific years, and a discount rate of 4.5% will be applied based on the economic feasibility evaluation guidelines of the Health Insurance Review and Assessment Service.

B. Analysis perspective

Societal perspective and restricted societal perspectives will be applied to include direct nonhealthcare, direct nonhealthcare, and lost productivity costs.

C. Sensitivity analysis

Deterministic sensitivity analysis will be conducted on all possible parameters and a tornado diagram will be presented. Probabilistic sensitivity analysis will be conducted using the distribution and representative values of all possible parameters. In addition, bootstrap using individual patient level data will be conducted to present the distribution of the economic feasibility evaluation effect index and 95% confidence intervals.

D. Assumptions for missing values

Last-Observation-Carried-Forward analysis method, a conservative method of underestimating missing values in cases where the result is expected to improve over time within the time horizon, is widely used to correct for missing values in clinical studies. Herein, the Last-Observation-Carried-Forward method will be used to assume missing values. In other words, if the patient is withdrawn from the study or completes treatment early, the last measured values will be used for all remaining visits to conduct analysis.

#### 2.9.1 Quality control and data monitoring

This study will be monitored by Korean Medicine Clinical Trial Center, a CRO company that consults with institutions conducting clinical trials to ensure compliance with protocol and K-GCP. During monitoring, crosschecks will be conducted with the evidence to ensure that the documents (trial master files, CRF, informed consent forms, and adverse events reports) are complete and clear.

#### 2.9.2 Research ethics approval

This trial has received complete ethical approval from the Ethics Committee of Catholic Kwandong University International St. Mary's Hospital (IS20OISE0085).

#### 2.9.3 Protocol amendments

The investigators who want to perform protocol amendments should first discuss it with the principal investigator, and they can change the protocol after obtaining approval from the IRB. However, when a dangerous situation occurs and immediate care is needed, the protocol change will be reported to the IRB at a later time.

#### 2.9.4 Post-trial care

If the patients experience unexpected accidents or injuries, the patients will receive appropriate medical care at the Catholic Kwandong University, International, St. Mary's Hospital. Additionally, appropriate compensation will be made by the insurance company, according to the patient compensation rules of the trial.

## 3 Discussion

Acupotomy is a treatment method of inserting a needle with a scalpel-shaped tip into a blood vessel, light muscle, or a tender point of the body. The needle then helps to detach the adhesion sites of muscles and ligaments with low energy and blood to treat relevant diseases. This treatment method is widely used in China and Korea. In particular, acupotomy is conducted to treat lumbosacral radiculopathy such as lumbar disk herniation and lumbospinal canal.^[4–13]^ In Korea, NBT is generally performed 3 times at two-week intervals for patients with lumbosacral radiculopathy. Patients who continuously experience pain while waiting for the next NBT treatment voluntarily undergo Korean medicine treatment such as acupotomy and acupuncture.

Although many patients with lumbosacral radiculopathy undergo Korean medicine treatment with NBT, there are no studies on combination treatment of acupotomy and NBT. Thus, there is a need for studies on the objective efficacy, safety, and economic feasibility of acupotomy and NBT combination treatment.

In this clinical study, patients whose pain will be improved by less than 50% and will have a NRS score greater than five after the first NBT will be included in order to select patients with weak response to NBT. This was to test the hypothesis that acupotomy, which has greater stimulation effects compared with acupuncture, will cause biomechanical effects, thereby leading to synergistic effects with the biochemical effects of NBT in patients with a weak response to NBT.

Objective evidence for the synergistic effects, safety, and economic feasibility of the combination treatment of NBT and acupotomy will suggest a new alternative treatment that can be used together with NBT, a standard treatment method. This would bring several direct benefits, such as reduced rate of surgeries to the patients.

## Author contributions

**Conceptualization:** Sooil Choi, Sukhee Park, Young-Soo Lim, Kwang-Sun Do, Sang Hyun Byun, Sang-Hoon Yoon.

**Funding acquisition:** Tae-Yong Park.

**Investigation:** Sooil Choi, Sukhee Park, Tae-Yong Park, Kwang-Sun Do, Jin-Hyun Lee.

**Resources:** Jin-Hyun Lee.

**Supervision:** Tae-Yong Park.

**Writing – original draft:** Tae-Yong Park, Jin-Hyun Lee.

**Writing – review & editing:** Sooil Choi.

## Supplementary Material

Supplemental Digital Content
