# Supplementary material for: A comparative study of a nerve block therapy with and without a deeply inserted acupotomy applied to hyeopcheok points for lumbosacral radiculopathy: Safety, effectiveness, cost-effectiveness (a randomized controlled, two-arm, parallel study, pilot study, assessor-blind)
Source: Medicine (Baltimore). 2022 Mar 4;101(9):e28983. doi: 10.1097/MD.0000000000028983 (PMC8896499; doi:10.1097/MD.0000000000028983)
Supplement: Supplemental Digital Content [file medi-101-e28983-s001.docx]

**Appendix**

**Appendix 1. Treatment points of acupotomy in the study**

*Essential treatment points*

Deeply insert to the Hyeopcheok acupoints (Ex-B2) corresponding to lumbar level of patients diagnosed with a disease related to lumbosacral radiculopathy by MRI or CT, and/or to the lumbar level related to the dermatome of the patient’s symptoms.

*Selectable treatment points*

Apply to the acupoints on the first line in bladder meridian (BL 22, 23, 24, 25, and 26) and/or Ashi acupoints around the affected area, according to the practitioner's judgment.
